# Book Review

**DOI:** 10.4269/ajtmh.2010.09-0472

**Published:** 2010-01

**Authors:** Duncan Chanda

**Affiliations:** *WHO-TDR TB/HAART Study University Teaching Hospital Lusaka, Zambia E-mail: drchandazm@yahoo.co.uk*

***Tuberculosis: A Comprehensive Clinical Reference.*** H. Simon Schaaf, Alimuddin I. Zumla, John Grange, Mario Raviglione, Wing Wai-Yew, Peter Donald, Madhukar Pai, and Jeffrey Starke, 2009. London, UK: WB Saunders Elsevier £119, ISBN 9781416039884 (hardback) and ISBN 9788131221518 (international edition).

Tuberculosis (TB) is today acknowledged as a global health catastrophe. In 1993, 111 years after Robert Koch's discovery of the cause of TB in 1882, the WHO declared TB “a global emergency.” In 2009, TB continues to kill 1.8 million every year and 5,000 people every day; that is one person every 20 seconds. Progress in TB management and control seems to have been lacking despite effective, inexpensive, and affordable chemotherapy being introduced over half a century ago. Over the past few years, interdisciplinary approaches and translational research on various aspects of TB is generating a large amount of data on conventional and molecular epidemiology, organism and host's interactions, new TB drugs and TB vaccine development, clinical management schedules for HIV-uninfected and HIV co-infected adults and children, development and evaluation of newer TB diagnostics, newer therapeutic regimens, and international standards of care for TB, among others. TB control programs around the world are faced with multidrug-resistant TB (MDR-TB) and extensively drug-resistant TB (XDR-TB), which are difficult and expensive to treat, threatening current efforts at TB control.

Over the years, education and training for TB health care workers has been neglected and sidelined. Educational material for improving clinic staff knowledge to carry out current global WHO TB diagnostics, treatment, and management recommendations are lacking. These are crucial in detecting and eliminating active cases of TB who spread the disease in the community. Current clinical TB textbooks on TB are very expensive; unaffordable in developing countries; outdated; predominantly written by UK/US authors; and do not have updated recommendations for treatment of HIV-infected patients with TB. New information on management of TB generated from recent clinical and basic science research is not filtered through to the relevant TB health care providers at points of care where TB guideline booklets, formulated decades ago by the WHO, are still in use. There is a great need to update and educate these TB carers on the latest developments.

For those of us with extensive experience in practicing medicine in a developing country, where poverty is rife and resources for TB care are scant, the quality of TB care delivered because of poor staff knowledge of the latest TB management recommendations hinders detection and effective treatment of active TB cases. The need for a comprehensive, up-to date textbook with the latest on TB management in HIV-infected and HIV-noninfected adults and children has been long overdue. A new TB textbook on the market has now fulfilled the needs described here. The textbook, entitled “Tuberculosis—A Comprehensive Clinical Reference” arises out of the global need to have a the latest developments in TB research and WHO-approved management guidelines incorporated into advice on day-to-day clinical practice for all TB care providers throughout the world. The editors of the textbook, global TB experts, Professors Zumla and Professor Simon Schaaf, teamed up with six international leading TB authorities as associate editors selected from across the globe: Professors John Grange (United Kingdom and Europe), Mario Raviglione (WHO, Geneva), Wing Wai Yew (Hong Kong), Jeffrey Starke (United States), Madhukar Pai (Canada), and Peter Donald (South Africa). This editorial team used 156 eminent TB-experienced authors from several continents (44 authors from Africa, 30 from the United States and Canada, 46 from Europe, and 8 from Asia) to write 107 chapters and 4 appendices constituting 1,008 pages to assemble this new textbook, a truly amazing and admirable achievement.

This unique, extraordinary, and comprehensive book is mainly a clinical one with emphasis on practical application at points of TB care. It covers every aspect of the clinical, epidemiologic, immunologic, laboratory, management, social science, point-of-care delivery, and translational research aspects of TB that are highly relevant to clinical practice and TB control in developing and western countries. This book has been skillfully crafted by the editors for it to be applicable for use all over the globe and extensively covers both adult and pediatric TB with equal emphasis and equal proportions. The bulk of the chapters are on clinical presentation, diagnosis, management, and well-illustrated system-based case histories of adult and childhood TB. All 107 chapters are intelligible, easily readable, written to the highest quality, and cover up-to-date, evidence-based information on the state-of-the-art of a wide spectrum of important TB subject areas. TB affecting every organ and tissue in the body is comprehensively covered in detail. It also comprehensively identifies and deals effectively with *MtB* and HIV co-infection issues and gives practical day-to-day management guidelines for point of TB care internationally.

In the short space provided here, it is difficult to do justice and highlight the superb features of this magnificent book. All chapters are highly illustrated (350 in the book), with elegantly presented figures, graphs, and tables adjunct to clinical, pathologic, and radiologic photos that show all minute details of the point being portrayed. The chapters review all current knowledge, gaps, and challenges ahead in various fields of TB. The book also deals with social and structural issues in TB and has very informative and useful appendices on conversion units for laboratory results, TB drug information, web sources on TB in general, and organizations supporting TB control and those providing research funding. This clinical textbook is aimed toward a variety of audiences, including general physicians, pulmonologists, TB and HIV clinic staff, medical assistants, nurse practitioners, TB program staff, medical students, postgraduates, technical staff, clinician scientists, policy makers, and donor agencies. The book opens with an emotional preface by His Grace, Archbishop Reverend Desmond Tutu, who himself had TB as a child. The book ends with a thought-provoking epilogue from Professors Zumla, Grange, and Schaaf, which introduces personal perspectives and optimistic caution.

Importantly, to make this clinically based textbook widely available and affordable in developing countries, the editors' commitment to the TB cause and the publishers have simultaneously published a cheaper discounted edition for purchase in poorer developing countries. This price will be reduced further as charities such as Bookpower and donor agencies promote this invaluable textbook. No doubt this textbook is the best treatise on TB available on the market and will educate millions of health workers for a long time to come. This book is an excellent example of what can be achieved by uniting the scientific global community in the fight against TB.

